# Efficacy and safety of Danhong injection on endothelial function and inflammatory factors after the percutaneous coronary intervention for coronary heart disease: A protocol of systematic review and meta-analysis of randomized controlled trials

**DOI:** 10.1097/MD.0000000000020783

**Published:** 2020-07-02

**Authors:** Shujuan Li, Shasha Duan, Yuzhen Ning, Hongmei Zhang, Qi Zheng

**Affiliations:** a Department of Emergency; b Department of Ultrasound, The Affiliated Hospital of Inner Mongolia Medical University, Huimin District, Hohhot, Inner Mongolia Autonomous Region, China.

**Keywords:** coronary heart disease, Danhong injection, endothelial function inflammatory factors, ercutaneous coronary intervention, postoperation, systematic review and meta-analysis

## Abstract

**Background:**

To systematically review the effects of Danhong injection on endothelial function and inflammatory factors after the percutaneous coronary intervention (PCI) for coronary heart disease (CHD) and to provide a basis for further research.

**Methods:**

Through computer retrieval, including PubMed, Embase, the Cochrane Library, CNKI, Wan Fang Data, VIP, SinoMed were retrieved on a computer. Randomized controlled trials (RCTs) on the effects of Danhong injection on endothelial function and inflammatory factors after PCI for CHD were collected in strict accordance with the pre-established inclusion and exclusion criteria. Chinese and English literatures in published from the establishment of each database to December 1, 2019, were retrieved by combining subject headings and free terms. Literatures were screened out by 2 researchers independently, and the risk of bias was assessed by 2 independent researchers by using the assessment tool for risk of bias as described Cochrane systematic reviewer's manual 5.1.0. Statistical analysis was performed by using Stata 14.0 software.

**Results:**

By collecting the existing evidence, this study would determine the effects of Danhong injection on endothelial function and inflammatory factors after PCI for CHD by meta-analysis.

**Conclusion:**

Through this study, we will draw a definite conclusion on whether Danhong injection has significant effects on endothelial function and inflammatory factors after PCI for CHD. This conclusion will provide practical and scientific guidance for the use of Danhong injection after PCI for CHD.

**PROSPERO registration number:**

PROSPERO CRD42020165568.

## 1 Introduction

Coronary arteriosclerotic heart disease, referred to as coronary heart disease (CHD), is caused by dyslipidemia, high blood pressure, impaired glucose tolerance, diabetes, smoking, etc. The clinical manifestations of the disease are thickening of the coronary artery wall and plaque formation in the coronary artery that narrows or obstructs the lumen, resulting in myocardial ischemia and hypoxia, which leads to CHD.^[1,2]^ The etiology of coronary atherosclerosis is still unclear. Related studies have shown that the formation of atherosclerosis is the joint result of many factors, including arterial wall cells, extracellular matrix (ECM), blood components (especially monocytes, platelets, and low-density lipoproteins), local hemodynamics, environment, and genetics. The key factors are vascular endothelial function damage and inflammatory response.^[3,4]^ The clinical symptoms and signs of CHD are often insidious in the early stage. During the progression of CHD, the imbalance between oxygen supply and consumption in myocardial cells caused by luminal stenosis leads to myocardial ischemia and hypoxia, which results in typical clinical symptoms. The deaths from CHD in western countries can account for 50% to 70% of the total deaths from heart diseases every year. In China, the fatality rate of CHD has roughly doubled over the past 2 decades, which is up to 1 million per year.^[5]^

Since the 1980s, percutaneous coronary intervention (PCI) has become one of the major treatments for CHD. This technology has achieved rapid development in China in the 21st century, and numerous clinical evidences have been gathered in related fields.^[6]^ However, a series of adverse events are also very likely to occur after PCI, such as no-reflow and slow flow, ischemia-reperfusion injury, perioperative myocardial injury, stent thrombosis, and restenosis. Studies have found that vascular endothelial function in patients with CHD is further reduced after PCI, which may be related to the damage to blood vessels, inflammation, and side effects of eluting drugs during the interventional procedures. Recent studies have shown that ischemia-reperfusion injury after PCI is closely related to inflammatory response, and high-sensitivity C-reactive protein (hs-CRP), matrix metalloproteinase-9 (MMP-9), and interleukin-6 (IL-6) play an important role in reperfusion damage after PCI. Ischemia-reperfusion injury after PCI is associated with myocardial injury, platelet activation, and increasing inflammatory factors, and is an important risk factor for cardiovascular adverse events.^[7,8]^ Therefore, related treatment methods for patients who received PCI is still an important direction in recent research.

Danhong injection is a traditional Chinese medicine injection prepared by extracting active ingredients from Salvia miltiorrhiza (Danshen) and safflower by modern pharmaceutical technology. Its main component is a water-soluble phenolic substance. Recent pharmacological research has found that in clinical practice, the functions of promoting blood circulation, and removing stasis of Danshen and safflower are commonly used to treat related diseases. Danshen can remove blood stasis, relieve pain, activate blood circulation, and stimulate meridians. The active ingredients in Danshen are tanshinone IIA, sodium sulfonate, and tanshinol.^[9,10]^ Current research on Danshen has found that tanshinone and safflower yellow can not only help dilate coronary arteries in human body, increase coronary blood flow, and improve the cardiac function, but also have a significant inhibitory effect on the coagulation process. That is to say, tanshinone and safflower yellow can interfere with platelet adhesion and aggregation, thus effectively improving hypercoagulability in the patient's body and preventing the occurrence of arterial thrombosis. This is of great significance for stabilizing the permeability of capillary walls. Also, under the effects of tanshinone and safflower yellow, the activities of oxide dismutase and glutathione peroxidase are increased, and their ability to resist free radical is improved, thereby protecting cells and controlling the occurrence of inflammatory reactions. Therefore, coronary heart disease can be prevented.^[11,12]^ However, considering the varying quality of related researches, there is the controversy over the effects of Danhong injection on endothelial function and inflammatory factors after PCI for CHD in clinical use among clinicians. Therefore, in this study we evaluated the effects of Danhong injection on endothelial function and inflammatory factors after PCI for CHD through systematic review, hoping to provide evidence for evidence-based medicine in further studies.

## 2 Methods and analysis

### 2.1 PROSPERO registration

The registration number is CRD42020165568 and registered on the PROSPERO.

### 2.2 Eligibility criteria

#### 2.2.1 Types of studies

Randomized controlled trials (RCT) with complete case data, whether based on blinding or concealed allocation, regardless of the language, time, and form of publication.

#### 2.2.2 Types of participants

Participants who met the diagnostic criteria for CHD after PCI and had no complications, regardless of sex, age, onset time, race, and nationality.

Inclusion criteria: Being confirmed by coronary angiography and meeting the diagnostic criteria for CHD in the “Diagnosis and Efficacy Criteria for Clinical Diseases”^[13]^; all patients were confirmed by coronary angiography to suffer from vascular stenosis >70% in at least one branch, or stenosis >50% in left main coronary artery; patients with the PCI indications specified in the “Guidelines for Percutaneous Coronary Intervention in China,” as suggested in examination^[14]^; meeting the requirements of randomized controlled trials.

Exclusion criteria: lacking full text through electronic and manual retrieval; repeatedly published literatures; studies with relevant information that cannot be extracted, such as interventions and outcome measures; literatures with abnormal data, statistical differences in baseline data, and incomplete data; inadequate trial design: literatures with incorrect random or statistical methods, such as randomly assigning patients according to outpatient numbers and order of visit; patients with severe primary diseases in brain, liver, kidney and endocrine, metabolic system or hematopoietic system. Patients treated with other traditional Chinese medicine.

#### 2.2.3 Types of interventions

The treatment timing of the 2 interventions was administration after PCI for ≥7 days without limiting the dosage, route of administration, and follow-up time.

Control group: conventional treatment after PCI. Conventional treatment included antiplatelet, anticoagulant, nitrates, β-blocker, calcium channel blocker, lipid-lowering drugs, etc.

Experimental group: Danhong injection combined with conventional treatment after PCI.

### 2.3 Types of outcome measures

#### 2.3.1 Primary outcomes

Measures related to endothelial function: brachial artery flow-mediated dilatation (FMD); nitric oxide (NO); endothelin (ET)/endothelin-1 (ET-1); von willebrand factor (vWF); homocysteine (Hcy), etc.

Measures of inflammatory factors: hs-CRP; IL-6; MMP-9; tumor necrosis factor-α (TNF-α).

#### 2.3.2 Secondary outcomes

Myocardial injury markers: cardiac troponin I (cTn I); creatine kinase-MB (CK-MB).

### 2.4 Data sources and search strategy

Through computer retrieval, 7 Chinese and English databases were retrieved, including CNKI, Wan Fang Data, VIP, CBMdisc, PubMed, The Cochrane Library, Embase. Randomized controlled trials (RCTs) on the effects of Danhong injection on endothelial function and inflammatory factors after PCI for CHD were collected in strict accordance with the pre-established inclusion and exclusion criteria. Chinese and English literatures in published from the establishment of each database to December 1, 2019 were retrieved by combining subject headings and free terms. Key words in English: “Danhong” OR “coronary heart disease” OR “percutaneous coronary intervention” OR “percutaneous coronary revascularization” OR “percutaneous coronary” OR “coronary revascularization” OR “coronary inter vention” or “PCI” or “end-otherelium, vasula” or “end-otherelial function” or “inflammation-f actors” OR “inflammatory cytokines.” Chinese search words (in Pinyin): “Danhong zhu she ye” OR “Guan xin bing” OR “jing pi guan zhuang dong mai jie ru zhi liao” OR “biao zhun qiu nang xue guan cheng xing shu” OR “jing pi guang Zhuang dong mai xue yun chong jian shu” OR “yan xing xi bao yin zi,” etc. References and journals in relevant fields in the studies included were also manually retrieved to obtain relevant randomized controlled studies of Danhong injection in the treatment of CHD after PCI. As an example, we searched the PubMed database, as shown in Table 1.

**Table 1:**
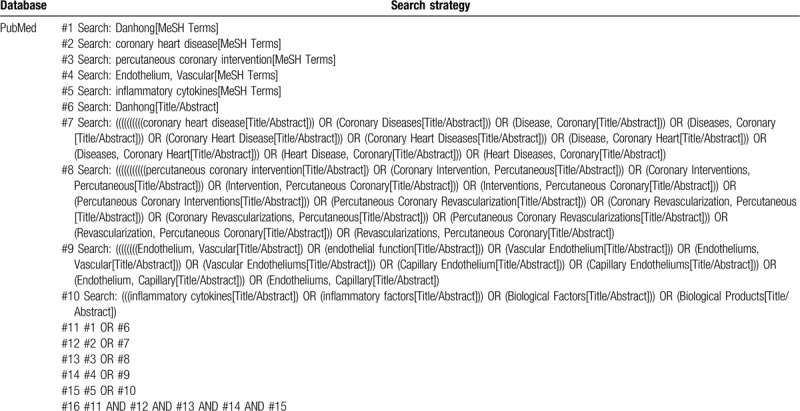
Search strategy of PubMed database.

### 2.5 Study selection and data extraction

**Figure 1:**
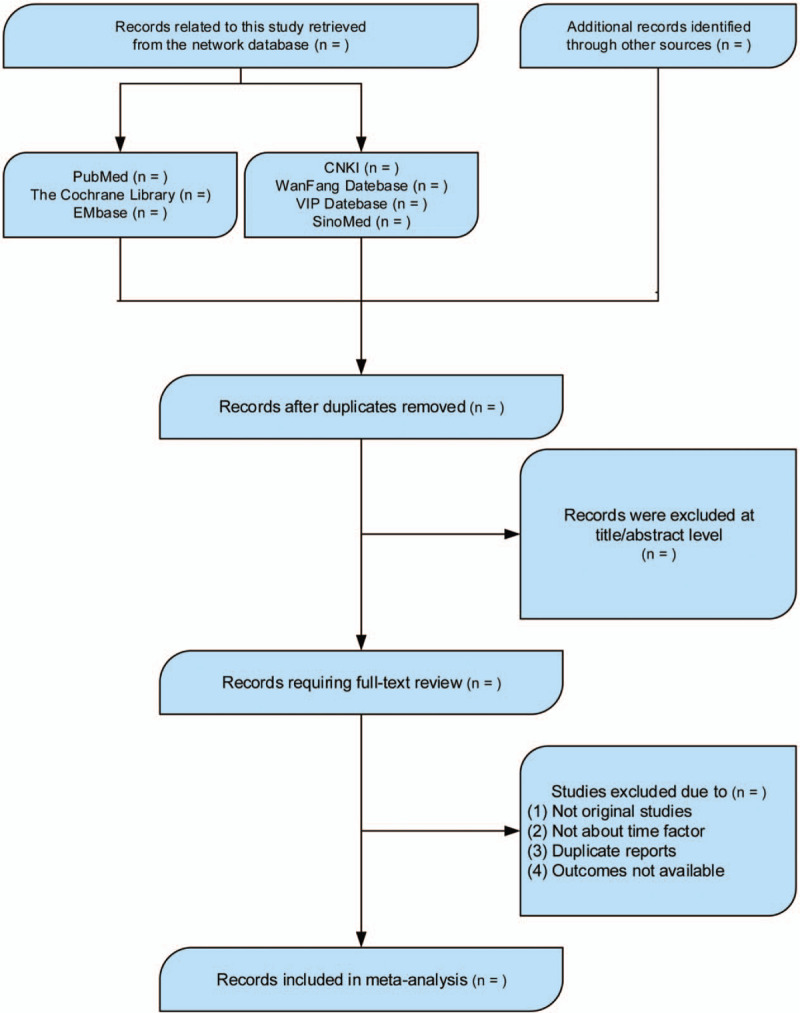
The PRISMA flow chart. PRISMA: Preferred Reporting Items for Systematic Reviews and Meta-Analyses.

Two researchers independently evaluated the quality of the literatures. Firstly, the titles and abstracts of the literatures searched were browsed, and the potentially qualified literatures were downloaded, and the full text was read. After contacting the original authors, supplementary information was provided in the literature that met the criteria. The reasons for exclusion were explained for literatures failing to meet the inclusion criteria. Data were independently extracted by 2 researchers, including the first author's name, publication time, paper title, disease name, number of samples in each group, intervention time, interventions in each group, outcome measures, risk of bias assessment, etc. After completion, 2 researchers cross-checked the results. Any inconsistency in the results was discussed or solved by consulting with a third investigator to reach consensus. The flow chart of literature screening is shown in Fig. 1.

### 2.6 Risk of bias assessment

The risk of bias in the included studies and clinical trials was assessed by using the risk of bias tool in the Cochrane Review Manager's Handbook.^[15,16]^ The following aspects were assessed: whether random sequence is generated properly and correctly (low risk, unknown risk, high risk); whether concealed allocation is proper and correct (low risk, unknown risk, high risk); whether the blinding method is implemented properly and correctly for subjects and researchers (low risk, unknown risk, high risk); whether the blinding method is implemented properly and correctly for evaluating the outcome of the study; whether outcome data are complete (low risk, unknown risk, high risk); whether research results are published selectively (low risk, unknown risk, high risk).

### 2.7 Data analysis and synthesis

RevMan 5.3 (The Nordic Cochrane Centre, The Cochrane Collaboration, London, United Kingdom) and Stata 14.0 (StataCorp LLC, Texas, USA) were used for statistical analysis in this study. The combined effect size was expressed as the relative ratio (RR), and an interval estimate was based on a 95% confidence interval (CI). Chi-square test was carried out to analyze heterogeneity among different studies. The significant level was set at *α* = 0.05, and *P* < .05 was considered as statistically significant. Heterogeneity was expressed as *I*^2^. *I*^2^ < 25% indicates lower heterogeneity, 25% ≤ *I*^2^ < 70% indicates moderate heterogeneity, and *I*^2^ > 70% indicates high heterogeneity. Subgroup analysis, sensitivity analysis, and other methods were used to detect the possible causes of clinical heterogeneity and statistical heterogeneity when *I*^2^ > 70% where necessary. When heterogeneity still existed after excluding the interference from the above-mentioned factors, Mantel-Haenszel (M-H) random effect model was used for combined analysis; descriptive analysis was made when heterogeneity was too large. Otherwise a fixed-effects model was used for combined analysis. A funnel plot was made to display publication bias, and Stata 14.0 software was used to make linear regression analysis by the Egger method to determine the symmetry.

### 2.8 Sensitivity analysis

Sensitivity analysis is to observe whether the synthesis result changes by altering some important factors and evaluate the stability of the result. The main methods include the one-by-one elimination method, subtraction method, alternative statistical methods, and effect model, among which the first 2 methods are most common. In the RevMan 5.3 software, the studies included was excluded one by one, and the effect size obtained were compared with the original results. A small change indicates that the sensitivity is low, and the results are robust and reliable. On the other hand, a significant difference between the 2 results suggests that the sensitivity is high, and the results are less robust and reliable.

### 2.9 Subgroup analysis

When there is relatively high heterogeneity between data, it is possible to perform subgroup analysis based on age, sex, and disease severity to find out the cause of heterogeneity. To avoid faulty analysis, we can perform a smaller unit-based analysis, namely subgroup analysis. However, due to the small number of studies, subgroup analysis may lead to false-positive results, which should be avoided in our study as much as possible.

### 2.10 Publication bias

Publication bias is a type of bias caused by researchers seeking too many positive results from trials. In the process of publication, positive results are more likely to be recognized and accepted by authoritative journals and more quickly to be published. Therefore, in some cases, researchers may make a positive result-oriented experiment design, or empathize positive results so much in writing their articles that negative results are not reported. In a meta-analysis, publication bias in the literatures included may also have an impact on the final results. At present, the funnel plot is the major method to detect publication bias in meta-analysis. In a funnel plot, the effect size is taken as the abscissa, while precision as the ordinate. Effect size refers to the ratio of the publication probability of positive results to the publication probability of negative results. Precision generally refers to the size of the samples included in the study. Literatures with a larger sample size often gather at the tip of the funnel plot. It is feasible to determine whether there is publication bias in literatures by observing the symmetry of the funnel plot.^[17]^

### 2.11 Ethics and dissemination

This ethical approval is not applicable to this study, and the research results will be published in professional academic journals.

## 3 Discussion

Cardiovascular disease (CVD) has become a significant public health problem that threatens global health. CHD, as an important part of CVD, is mainly caused by atherosclerosis that leads to organic obstruction or stenosis of the coronary artery, resulting in insufficient coronary blood supply, myocardial hypoxia and ischemia, and even myocardial infarction. The popularity and development of minimally invasive technology have significantly improved the treatment efficiency of patients with CHD, and coronary revascularization has become an important method for coronary intervention.^[18]^ PCI has been rapidly popularized since its inception, and now it has become the first choice for the clinical treatment of CVD. The way PCI restores blood flow is to dilate the patient's narrow coronary arteries by balloon dilation and stent implantation. However, surgery may stimulate vascular endothelial damage and release a large number of inflammatory factors, inducing local inflammatory reaction. Therefore, the process of PCI may cause certain damage to the patient's vascular endothelial cells and then trigger systemic or local inflammatory reactions. Vasospasm and blood stasis caused by such inflammatory reactions may also affect vascular function and structural stability, leading to restenosis after surgery. Furthermore, small clots may detach in some patients following PCI, resulting in the occurrence of distal embolism, and then causing secondary stenosis in blood vessels and triggering angina. Hence, in addition to conventional depressurization, anticoagulant and lipid-lowering according to the actual needs of patients after PCI, the damage to vascular endothelial cells and inflammatory reactions caused by minimally invasive surgery should also be considered.^[19]^

Angina pectoris is often classified as “heartache” and “chest obstruction” in traditional Chinese medicine. It is thought that the cause of such disease generally involves blood stasis and heart vessel blockage stasis. Therefore, the method of promoting blood circulation and removing blood stasis is unusually used in the treatment process. Chinese patent medicines have a unique regulatory effect on vascular endothelial function, and the prevention and treatment of the cardiovascular disease can be explored accordingly.^[20,21]^ Danhong injection has the effects of promoting blood circulation, relieving meridians, activating blood, and dissolving stasis. Salvianolate in Danshen can reduce the levels of serum resistin and CRP, increase the level of adiponectin, and protect the vascular endothelial function. It can also improve the antioxidant capacity of myocardial tissue by eliminating oxygen free radicals in ischemic myocardium. On the other hand, safflower yellow in safflower can inhibit platelet adhesion and aggregation, activate vascular endothelial cells to release prostaglandin I2 (PGI2), and correct the disequilibrium of Thromboxane A2 (TXA2)/PGI2 in peripheral circulation.^[22,23]^

Although related literatures have reported the research of Danhong injection on endothelial function and inflammatory factors after PCI for CHD, evidence for evidence-based medicine is still lacking. Therefore, the development of this research is very necessary.

## Author contributions

**Conceptualization:** Qi Zheng, Shujuan Li.

**Data curation:** Shujuan Li, Yuzhen Ning.

**Formal analysis:** Shasha Duan, Hongmei Zhang.

**Funding acquisition:** Shujuan Li.

**Methodology:** Shujuan Li, Shasha Duan.

**Project administration:** Qi Zheng.

**Software:** Shujuan Li, Shasha Duan, Yuzhen Ning, Hongmei Zhang.

**Supervision:** Qi Zheng.

**Writing – original draft:** Shujuan Li, Shasha Duan, Yuzhen Ning, Hongmei Zhang.

**Writing – review & editing:** Qi Zheng.
